# Down-regulation of *OPA1* in patients with primary open angle glaucoma

**Published:** 2011-04-27

**Authors:** Thomas M. Bosley, Ali Hellani, George L. Spaeth, Jonathan Myers, L. Jay Katz, Marlene R. Moster, Barry Milcarek, Khaled K. Abu-Amero

**Affiliations:** 1 Ophthalmic Genetics Laboratory and Department of Ophthalmology, College of Medicine, King Saud University, Riyadh, Saudi Arabia; 2 Division of Neurology, Cooper University Hospital, Camden, NJ; 3 PGD Laboratory, Saad Specialist Hospital, Al-Khobar, Saudi Arabia; 4 William and Anna Goldberg Glaucoma Service, Wills Eye Hospital, Thomas Jefferson University, Philadelphia, PA; 5 Research Department, Cooper University Hospital, Camden, NJ; 6 Department of Ophthalmology, University of Florida-College of Medicine, Jacksonville, FL

## Abstract

**Purpose:**

Heterozygous optic atrophy type1 (*OPA1*) mutations are responsible for dominant optic atrophy, and the down regulation of *OPA1* expression in patients with Leber hereditary optic neuropathy may imply that Opa1 protein levels in mitochondria play a role in other spontaneous optic neuropathies as well. Mitochondrial and metabolic abnormalities may put the optic nerve at risk in primary open angle glaucoma (POAG), and this preliminary study was designed to investigate whether altered *OPA1* expression might be present in the progressive optic neuropathy of POAG.

**Methods:**

Patients were eligible for inclusion if they met standard clinical criteria for POAG, including age greater than 40 years, intraocular pressure ≥ 21 mmHg in at least one eye before treatment, normal-appearing anterior chamber angles bilaterally on gonioscopy, and optic nerve injury characteristic of POAG. RNA was extracted from leukocytes and converted to cDNA by reverse transcriptase enzyme, and real time PCR was used to assess expression levels of *OPA1* and the β-globulin (*HBB*) housekeeping gene. The ratio of *OPA1* expression to *HBB* expression (*OPA1/HBB*) for POAG patients was compared to that of controls and to clinical characteristics of POAG patients.

**Results:**

Forty-three POAG patients and 27 controls were completely phenotyped with a full ophthalmologic examination and static perimetry. Mean age (POAG 67.9 years; controls 61.8 years) and sex (POAG 26 males/17 females; controls 11/16) were similar for the two groups. Mean *OPA1/HBB* of POAG patients (1.16, SD 0.26) was 18% lower than controls (1.41, SD 0.50), and this difference was statistically significant (p≤0.021). *OPA1* expression differed between the groups (p≤0.037), but *HBB* expression did not differ (p≤0.24). *OPA1/HBB* was not correlated with any clinical feature of POAG patients.

**Conclusions:**

Transcriptional analysis of peripheral blood leucocytes is a limited model system for studying the consequences of mitochondrial abnormalities in the optic nerve. Nevertheless, *OPA1* is known to affect mitochondrial stability and has now been implicated in several spontaneous optic neuropathies. Decreased *OPA1* expression in POAG patients is another indication that mitochondrial function, and possibly mitochondrially-induced apoptosis, may play a role in the development of POAG.

## Introduction

Glaucoma is one of the leading causes of blindness worldwide [1] with a prevalence of over 2% in individuals older than 40 years [2]. Primary open angle glaucoma (POAG) is the most common type of glaucoma in Western countries and has risk factors that include elevated intraocular pressure (IOP) and age [3], but these factors do not predict the presence or degree of visual loss [4]. Up to half of all patients with POAG have a positive family history, and the risk of POAG is increased 3–9 times in first-degree relatives of POAG patients [2,5]. In addition, a maternal family history of POAG is 6–8 times more likely than a paternal family history [6-8]. These observations suggest that genetic factors may contribute to POAG, with a mitochondrial component being particularly likely [1,9-11].

Mutations in the optic atrophy type1 (*OPA1*) gene are unequivocally involved in the neuropathology of dominant optic atrophy (DOA) [12,13]. The phenotype of DOA differs from that of POAG, but DOA is sometimes misdiagnosed as normal tension glaucoma in clinical practice [14]. In addition, certain *OPA1* polymorphisms have been linked to an increased risk of POAG in some, but not all, ethnic populations [15-18] and with the normal tension variant of POAG [17,19]. Considered together, these observations suggest that *OPA1* may be involved in the development of POAG.

## Methods

Patients were evaluated in the Glaucoma Service at the Wills Eye Institute and enrolled after examination by a glaucoma specialist. Patients were eligible for inclusion if they met the following clinical criteria for POAG [20-23]: age greater than 40 years; intraocular pressure (IOP) ≥21 mmHg in one or both eyes before initiation of glaucoma treatment; normal-appearing, open anterior chamber angles bilaterally by gonioscopy; optic nerve appearance characteristic of the optic discs typically observed in primary open-angle glaucoma (with localized narrowing or absence of the neuro- retinal rim, with the amount of cupping exceeding the amount of pallor of the rim, and with asymmetric cupping of the optic discs in the two eyes); and static visual field (Humphrey Field Analyzer II, Carl Zeiss Meditec, Inc., Dublin, CA; using a full threshold 24–2 program) abnormalities typical of glaucoma (as per Advanced Glaucoma Intervention Study criteria [24]). There had to be good agreement between the appearance of the optic disc and the visual field. Exclusion criteria included historical, neuroimaging, or biochemical evidence of another possible optic neuropathic process affecting either eye, significant visual loss in both eyes not associated with glaucoma, or choosing not to participate. This research adhered to the tenets of the Declaration of Helsinki, and all patients and controls signed an informed consent approved by the Wills Eye Institute institutional review board.

All control subjects (frequently spouses of patients) had full ophthalmologic examinations and static perimetry. Each had IOPs that were below 21 mmHg and symmetric in the two eyes, normal anterior chambers, optic discs that were normal and symmetric in appearance, entirely normal static perimetry OU, and no prior history of glaucoma. All controls had static perimetry performed in the same fashion as POAG patients.

A two-step semi-quantitative RT–PCR method was used to measure gene expression levels of *OPA1* and β-globulin (*HBB*) in POAG patients and controls. Random hexameres were used as primers in the first step of cDNA synthesis. Total RNA (1 μg) was combined with 0.5 μg primers, 200 μM dNTPs, and sterile Milli-Q water and preheated at 65 °C for 2 min to denature secondary structures. The mixture was then cooled rapidly to 20 °C and then 10 μl 5× RT Buffer, 10 mM dithiothreitol, and 200 U Moloney Murine Leukemia Virus Reverse Transcriptase were added for a total volume of 50 μl. The RT mix was incubated at 37 °C for 90 min then stopped by heating at 95 °C for 5 min. The cDNA stock was stored at −20 °C.

**Figure 1:**
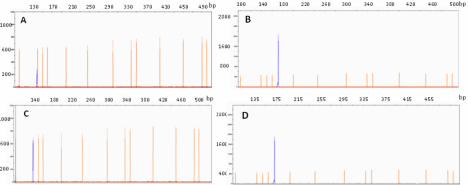
Fluorescent PCR amplification. Capillary electrophoresis showing fluorescent PCR amplification of (A) the OPA1 gene (140 bp; blue peak) and (B) the HBB gene (175 bp) for a POAG patient. Similarly, (C) shows OPA1 amplification and (D) shows HBB amplification for a control individual. The x-axis represents peak size in base pairs (bp) and the y-axis the signal intensity. The area under the peak (not shown here) corresponds to gene expression levels and was measured by the GeneMapper software (Applied Biosystems). Sizing of the peaks was done by running the 500 pb LIZ size-ladder (peaks in orange) with the samples.

Relative RT–PCR was performed to measure gene expression of *OPA1* and *HBB* according to standard guidelines [25]. Primer sequences and optimal PCR annealing temperatures (ta) are listed in Table 1. Primer sequences were designed to span intron regions to insure that no false positive PCR fragments would be generated from pseudogenes and contaminate genomic DNA. In addition, all forward PCR primers were labeled with fluorescein (6-FAM), making quantitation more accurate. Polymerase chain reactions were performed using 100 ng of cDNA, 5 pmoles of each oligonucleotide primer, 200 μM of each dNTP, 1 unit of HotStar Taq-polymerase (Qiagen, Valencia, CA) and 1× PCR buffer in a 20 μl volume. The PCR program initially started with a 95 °C denaturation for 5 min, followed by 25 cycles of 95 °C for 1 min, ta °C for 45 s, and 72 °C for 1 min. Linear amplification range for each gene was tested on the adjusted cDNA, and 25 cycles were found to be optimal for both *OPA1* and *HBB*. The PCR samples were electrophoresed on the 3130xl Genetic Analyzer (Applied Biosystems, Foster City, CA). Figure 1 shows representative chromatograms for a POAG patient and a control individual in which the size and the intensity of the fluorescence peaks are illustrated for *OPA1* and *HBB* amplification.

**Table 1:** Primer sequences and annealing temperatures.

**Primer name**	**Primer sequence**	**Annealing temperature (°C)**
*HBB*-F	(6-FAM) AGC CTC GCC TTT GCC GA	57
*HBB*-R	CTG GTG CCT GGG GCG	
*OPA1*-F	(6-FAM) TGT GAG GTC TGC CAG TCT TTA	58
*OPA1*-R	TGT CCT TAA TTG GGG TCG TTG	

F=forward; R=reverse.

The promoter region of the *OPA1* gene was screened for polymorphisms in all patients and controls using the forward (5′-CCT TTC CCA TCT GAT CCT CA-3′) and reverse (5′-CAG GAA TGA CCC AGG AAG TG-3′) primers to amplify a region extending almost 700 bp immediately upstream of the transcription initiation site, where the promoter region of *OPA1* is believed to be located [13].

### Statistical analysis

Absolute RT–PCR values were used to calculate a ratio of the *OPA1* peak area in the selected linear amplification cycle divided by that of *HBB*, creating an *OPA1/HBB* ratio. All clinical and genetic data were analyzed using SPSS v 16.02 (IBM Corporation, Somers, NY) and SAS v 9.2 (SAS Corporation, Cary, NC).

## Results

Age (POAG patients 67.0 years; controls 61.8 years; p≤0.06) and sex (POAG 26 males/17 females; controls 11/16; p≤0.11) of the 43 unrelated POAG patients were similar to the control individuals, but ethnicity differed between the POAG group (21 Caucasian/22 African American) and the control group (23 Caucasian/4 African American; p≤0.002).

Mean *OPA1* expression values were significantly lower in POAG patients than in controls (p≤0.037) but *HBB* expression did not differ between the groups (p≤0.34; Table 2). The *OPA1/HBB* ratio was also significantly lower, by 18%, in POAG patients than in controls (p≤0.021).

**Table 2:** Gene expression in POAG patients and controls.

**Measurement**	**POAG mean**	**Control mean**	**p≤**
*OPA1* expression; mean (SD)	116714 (22220)	137761 (47076)	0.04
*HBB* expression; mean (SD)	104489 (33188)	98164 (7707)	0.24
*OPA1*/*HBB*; mean (SD)	1.16 (0.26)	1.41 (0.50)	0.02
*OPA1* expression in Caucasians; mean (SD)	112979 (15699)	133460 (45261)	0.04
*HBB* expression in Caucasians; mean (SD)	108507 (47291)	98462 (7931)	0.34
*OPA1*/*HBB* in Caucasians; mean (SD)	1.12 (0.25)	1.37 (0.50)	0.04

POAG=primary open angle glaucoma; SD=standard deviation.

Differences in the *OPA1/HBB* ratio persisted even when the evaluated groups were restricted to Caucasian patients and controls (p≤0.04) to remove the potential effect of ethnic bias. Similarly, mean *OPA1* expression values were significantly lower in Caucasian POAG patients than in Caucasian controls (p=0.04), but *HBB* expression did not significantly differ between these groups (p≤0.34; Table 2).

Within the POAG group, the *OPA1/HBB* ratio was not significantly associated with age, sex, ethnicity, visual acuity, maximum intraocular pressure, vertical cup-to-disk ratio, static perimetry mean deviation, or static perimetry pattern standard deviation (Table 3). However, power calculations indicate power ≤80% on these tests, leaving open the possibility of false negative type II statistical errors.

**Table 3:** Correlation between *OPA1*/*HBB* and clinical parameters

**Parameter**	**Value**	**p≤**
Age in years; mean (SD)	67.93 (10.55)	0.90
Sex (M:F)	26:17	0.10
Ethnicity (C:AA)	21:22	0.27
Visual acuity OD (mean)	~20/40	0.74
Visual acuity OS (mean)	~20/30	0.56
Maximum IOP OD (mmHg)	29.4 (6.3)	0.07
Maximum IOP OS (mmHg)	29.7 (6.9)	0.13
Vertical c/d ratio OD; mean (SD)	0.78 (0.21)	0.84
Vertical c/d ratio OS; mean (SD)	0.76 (0.20)	0.99
MD OD; mean (SD)	−15.46 (10.28)	0.28
MD OS; mean (SD)	−13.27 (10.37)	0.33
PSD OD; mean (SD)	7.52 (4.51)	0.74
PSD OS; mean (SD)	7.01 (4.33)	0.92

SD=standard deviation; M=male; F=female; C=Caucasian; AA=African American; OD=right eye; OS=left eye; IOP=intraocular pressure; mmHg=mm mercury; c/d=cup to disk; MD=Humphrey visual field mean deviation; PSD=Humphrey visual field pattern standard deviation.

Neither POAG patients nor controls had polymorphisms in the promoter region after reading and aligning all sequences with the *OPA1* promoter reference sequence described previously [13].

## Discussion

The 43 patients reported here met rigorous clinical criteria for POAG [20-23] with elevated IOP, normal anterior chamber, and evidence on funduscopic exam and visual fields of glaucomatous optic nerve damage. They did not have evidence by clinical criteria of other types of glaucoma or alternative causes of optic nerve injury. None had dysmorphism or an obvious genetic syndrome. They were compared to 27 control individuals in whom POAG and other evidence of optic nerve damage were carefully excluded.

*OPA1* expression and the *OPA1/HBB* ratio were both significantly lower in POAG patients than in controls (Table 2). POAG patients and controls were well matched for age and sex. The POAG group included more African Americans than the control group; however, *OPA1* expression and *OPA1/HBB* ratio were still significantly smaller in POAG patients when both groups were restricted only to Caucasians. No POAG patient had a mutation or polymorphism of the *OPA1* gene or the *OPA1* promoter region that might explain an alteration of expression.

Across all POAG patients, the *OPA1/HBB* ratio was not correlated with any demographic measure (e.g., age, sex, ethnicity, or age at diagnosis) or any standard measure of POAG severity (e.g., visual acuity, vertical cup-to-disk ratio, or measures of static perimetry abnormality; Table 3). The results of this study, therefore, imply that decreased *OPA1* expression, and possibly decreased Opa1 protein levels, may contribute to the occurrence of POAG. They do not suggest that *OPA1* expression is related to POAG severity, although this point may only be proven definitely by evaluating larger numbers of patients for longer periods of time.

The *OPA1* gene was first identified as the gene responsible for DOA [12,13] a decade ago. It codes for a dynamin-like GTPase protein (Opa1 protein) found in a polymeric structure in the inner mitochondrial membrane that has multiple distinct roles [26,27] primarily related to maintaining a highly interconnected mitochondrial network [28], regulating mitochondrial bioenergetic output through a possible effect on the assembly and stability of Complex I and IV subunits [29,30], and sequestering pro-apoptotic small molecules within the mitochondrial cristae spaces [31,32]. Maintenance of mitochondrial DNA (mtDNA), at least in certain settings [33], is a more recently recognized role.

*OPA1* abnormalities are now recognized in several spontaneous optic neuropathies. Heterozygous mutations in *OPA1* are responsible for approximately 60% of DOA cases [34,35], and it is possible that another 20% of DOA cases are associated with large-scale rearrangements of the entire *OPA1* coding region [36]. We reported previously that *OPA1* expression was decreased in patients with LHON and the 11778 primary LHON mutation [37]. Certain *OPA1* polymorphisms have been described in association with an increased risk of developing POAG or normal tension glaucoma in some, but not all, populations studied [15-17].

The results of this study raise the possibility that decreased *OPA1* expression may play a role in in occurrence of POAG in several ways. Down-regulation of *OPA1* in HeLa cells using specific small interfering RNA molecules caused fragmentation of the mitochondrial network with dissipation of the mitochondrial membrane potential and a drastic disorganization of the cristae [38]. These changes were followed by cytochrome c release and caspase-dependent apoptotic nuclear events. Mitochondria in this setting likely do not produce energy optimally and may either cause or be vulnerable to oxidative stress [39]. Decreased *OPA1* expression in POAG patients may contribute to retinal ganglion cell apoptosis as one primary mechanism of optic nerve damage. Decreased ATP production and/or increased oxidative stress may contribute to this process, particularly as mitochondria work less well in an aging individual.

This study has several practical limitations. The number of individuals studied was adequate to confirm statistically significant differences in *OPA1* expression and *OPA1/HBB* ratio between POAG patients and controls. However, the lack of correlation between *OPA1/HBB* ratio and various clinical parameters within the POAG group may be subject to type II statistical errors because of inadequate power. Transcriptional analysis of peripheral blood leucocytes is not an ideal model system for evaluating nuclear gene expression in optic nerve tissue, which will rarely be available in glaucoma patients. However, peripheral blood cells inherit the same genetic information as retinal ganglion cells, and leukocyte gene expression reflects pathologically important gene expression changes in certain other neurologic diseases [40-42]. The population studied was predominantly Caucasian and African-American, and different results might be obtained in other ethnicities.

The patients reported here have clinically definite POAG with a modest, statistically significant decrease in *OPA1* expression even after normalization for expression of *HBB*, a housekeeping gene. *OPA1* plays an important role in mitochondrial structure and function, including mitochondrially-induced apoptosis. It has been implicated in the pathogenesis of several other spontaneous optic neuropathies, and this study suggests that *OPA1* expression may also play a role in the development of POAG. If these results are confirmed in additional patient groups and other ethnicities, it is possible that methods might be found to interfere with POAG development by manipulation of *OPA1* expression or other mitochondrial parameters.

## References

[1] Quigley HA (1996). Number of people with glaucoma worldwide.. Br J Ophthalmol.

[2] Tielsch JM, Sommer A, Katz J, Royall RM, Quigley HA, Javitt J (1991). Racial variations in the prevalence of primary open-angle glaucoma. The Baltimore Eye Survey.. JAMA.

[3] Gherghel D, Hosking SL, Orgul S (2004). Autonomic nervous system, circadian rhythms, and primary open-angle glaucoma.. Surv Ophthalmol.

[4] Spaeth GL (1994). A new classification of glaucoma including focal glaucoma.. Surv Ophthalmol.

[5] Wilson MR, Hertzmark E, Walker AM, Childs-Shaw K, Epstein DL (1987). A case-control study of risk factors in open angle glaucoma.. Arch Ophthalmol.

[6] Morgan RW, Drance SM (1975). Chronic open-angle glaucoma and ocular hypertension. An epidemiological study.. Br J Ophthalmol.

[7] Shin DH, Becker B, Kolker AE (1977). Family history in primary open-angle glaucoma.. Arch Ophthalmol.

[8] Charliat G, Jolly D, Blanchard F (1994). Genetic risk factor in primary open-angle glaucoma: a case-control study.. Ophthalmic Epidemiol.

[9] Tielsch JM, Katz J, Sommer A, Quigley HA, Javitt JC (1994). Family history and risk of primary open angle glaucoma. The Baltimore Eye Survey.. Arch Ophthalmol.

[10] Quigley HA, Broman AT (2006). The number of people with glaucoma worldwide in 2010 and 2020.. Br J Ophthalmol.

[11] Abu-Amero KK, Morales J, Bosley TM (2006). Mitochondrial abnormalities in patients with primary open-angle glaucoma.. Invest Ophthalmol Vis Sci.

[12] Alexander C, Votruba M, Pesch UE, Thiselton DL, Mayer S, Moore A, Rodriguez M, Kellner U, Leo-Kottler B, Auburger G, Bhattacharya SS, Wissinger B (2000). OPA1, encoding a dynamin-related GTPase, is mutated in autosomal dominant optic atrophy linked to chromosome 3q28.. Nat Genet.

[13] Delettre C, Lenaers G, Griffoin JM, Gigarel N, Lorenzo C, Belenguer P, Pelloquin L, Grosgeorge J, Turc-Carel C, Perret E, Astarie-Dequeker C, Lasquellec L, Arnaud B, Ducommun B, Kaplan J, Hamel CP (2000). Nuclear gene OPA1, encoding a mitochondrial dynamin-related protein, is mutated in dominant optic atrophy.. Nat Genet.

[14] Buono LM, Foroozan R, Sergott RC, Savino PJ (2002). Is normal tension glaucoma actually an unrecognized hereditary optic neuropathy? New evidence from genetic analysis.. Curr Opin Ophthalmol.

[15] Aung T, Ocaka L, Ebenezer ND, Morris AG, Krawczak M, Thiselton DL, Alexander C, Votruba M, Brice G, Child AH, Francis PJ, Hitchings RA, Lehmann OJ, Bhattacharya SS (2002). A major marker for normal tension glaucoma: association with polymorphisms in the OPA1 gene.. Hum Genet.

[16] Aung T, Ocaka L, Ebenezer ND, Morris AG, Brice G, Child AH, Hitchings RA, Lehmann OJ, Bhattacharya SS (2002). Investigating the association between OPA1 polymorphisms and glaucoma: comparison between normal tension and high tension primary open angle glaucoma.. Hum Genet.

[17] Powell BL, Toomes C, Scott S, Yeung A, Marchbank NJ, Spry PG, Lumb R, Inglehearn CF, Churchill AJ (2003). Polymorphisms in OPA1 are associated with normal tension glaucoma.. Mol Vis.

[18] Woo SJ, Kim DM, Kim JY, Park SS, Ko HS, Yoo T (2004). Investigation of the association between OPA1 polymorphisms and normal-tension glaucoma in Korea.. J Glaucoma.

[19] Yu-Wai-Man P, Stewart JD, Hudson G, Andrews RM, Griffiths PG, Birch MK, Chinnery PF (2010). OPA1 increases the risk of normal but not high tension glaucoma.. J Med Genet.

[20] Spaeth GL (2007). Prognostic factors for progression of visual field damage in patients with normal-tension glaucoma.. Jpn J Ophthalmol.

[21] Eid TM, Spaeth GL, Bitterman A, Steinmann WC (2003). Rate and amount of visual loss in 102 patients with open-angle glaucoma followed up for at least 15 years.. Ophthalmology.

[22] Bayer A, Harasymowycz P, Henderer JD, Steinmann WG, Spaeth GL (2002). Validity of a new disk grading scale for estimating glaucomatous damage: correlation with visual field damage.. Am J Ophthalmol.

[23] Read R, Spaeth G (1974). The natural history of cup progression and some specific disc field correlations.. Trans Am Acad Ophthalmol Otolaryngol.

[24] Kim J, Dally LG, Ederer F, Gaasterland DE, VanVeldhuisen PC, Blackwell B, Sullivan EK, Prum B, Shafranov G, Beck A, Spaeth GL, Investigators A (2004). The Advanced Glaucoma Intervention Study (AGIS): 14. Distinguishing progression of glaucoma from visual field fluctuations.. Ophthalmology.

[25] Bustin SA, Beaulieu JF, Huggett J, Jaggi R, Kibenge FS, Olsvik PA, Penning LC, Toegel S (2010). MIQE precis: Practical implementation of minimum standard guidelines for fluorescence-based quantitative real-time PCR experiments.. BMC Mol Biol.

[26] Davies V, Votruba M (2006). Focus on molecules: the OPA1 protein.. Exp Eye Res.

[27] Lenaers G, Reynier P, Elachouri G, Soukkarieh C, Olichon A, Belenguer P, Baricault L, Ducommun B, Hamel C, Delettre C (2009). OPA1 functions in mitochondria and dysfunctions in optic nerve.. Int J Biochem Cell Biol.

[28] Chan DC (2007). Mitochondrial dynamics in disease.. N Engl J Med.

[29] Chevrollier A, Guillet V, Loiseau D, Gueguen N, de Crescenzo MA, Verny C, Ferre M, Dollfus H, Odent S, Milea D, Goizet C, Amati-Bonneau P, Procaccio V, Bonneau D, Reynier P (2008). Hereditary optic neuropathies share a common mitochondrial coupling defect.. Ann Neurol.

[30] Zanna C, Ghelli A, Porcelli AM, Karbowski M, Youle RJ, Schimpf S, Wissinger B, Pinti M, Cossarizza A, Vidoni S, Valentino ML, Rugolo M, Carelli V (2008). OPA1 mutations associated with dominant optic atrophy impair oxidative phosphorylation and mitochondrial fusion.. Brain.

[31] Frezza C, Cipolat S, Martins de Brito O, Micaroni M, Beznoussenko GV, Rudka T, Bartoli D, Polishuck RS, Danial NN, De Strooper B, Scorrano L (2006). OPA1 controls apoptotic cristae remodeling independently from mitochondrial fusion.. Cell.

[32] Olichon A, Landes T, Arnaune-Pelloquin L, Emorine LJ, Mils V, Guichet A, Delettre C, Hamel C, Amati-Bonneau P, Bonneau D, Reynier P, Lenaers G, Belenguer P (2007). Effects of OPA1 mutations on mitochondrial morphology and apoptosis: relevance to ADOA pathogenesis.. J Cell Physiol.

[33] Yu-Wai-Man P, Sitarz KS, Samuels DC, Griffiths PG, Reeve AK, Bindoff LA, Horvath R, Chinnery PF (2010). OPA1 mutations cause cytochrome c oxidase deficiency due to loss of wild-type mtDNA molecules.. Hum Mol Genet.

[34] Newman NJ, Biousse V (2004). Hereditary optic neuropathies.. Eye (Lond).

[35] Yu-Wai-Man P, Griffiths PG, Hudson G, Chinnery PF (2009). Inherited mitochondrial optic neuropathies.. J Med Genet.

[36] Fuhrmann N, Alavi MV, Bitoun P, Woernle S, Auburger G, Leo-Kottler B, Yu-Wai-Man P, Chinnery P, Wissinger B (2009). Genomic rearrangements in OPA1 are frequent in patients with autosomal dominant optic atrophy.. J Med Genet.

[37] Abu-Amero KK, Jaber M, Hellani A, Bosley TM (2010). Genome-wide expression profile of LHON patients with the 11778 mutation.. Br J Ophthalmol.

[38] Olichon A, Baricault L, Gas N, Guillou E, Valette A, Belenguer P, Lenaers G (2003). Loss of OPA1 perturbates the mitochondrial inner membrane structure and integrity, leading to cytochrome c release and apoptosis.. J Biol Chem.

[39] Tezel G (2006). Oxidative stress in glaucomatous neurodegeneration: mechanisms and consequences.. Prog Retin Eye Res.

[40] Stamova B, Xu H, Jickling G, Bushnell C, Tian Y, Ander BP, Zhan X, Liu D, Turner R, Adamczyk P, Khoury JC, Pancioli A, Jauch E, Broderick JP, Sharp FR (2010). Gene expression profiling of blood for the prediction of ischemic stroke.. Stroke.

[41] Liao IH, Sharp FR (2010). Tourette syndrome: gene expression as a tool to discover drug targets.. Neurotherapeutics.

[42] Tang Y, Gilbert DL, Glauser TA, Hershey AD, Sharp FR (2005). Blood gene expression profiling of neurologic diseases: a pilot microarray study.. Arch Neurol.

